# Pharmacy professionals’ views regarding the future of NHS patient medicines helpline services: a multimethod qualitative study

**DOI:** 10.1186/s12913-021-06144-6

**Published:** 2021-02-12

**Authors:** Matt Williams, Abbie Jordan, Jenny Scott, Matthew D. Jones

**Affiliations:** 1 Department of Pharmacy & Pharmacology, University of Bath, 5 West, Claverton Down, BA2 7AY, Bath, UK; 2 Department of Psychology & Centre for Pain Research, University of Bath, Bath, UK

**Keywords:** Patient medicines helplines, National Health Service, Medicines information, Drug information, Hospital pharmacy, Hospital discharge, Medicines‐related errors, Qualitative, Multimethod, Thematic analysis

## Abstract

**Background:**

Patient medicines helpline services (PMHS) have been established at some National Health Service (NHS) hospitals, to provide patients with post-discharge medicines-related support. However, findings suggest that many PMHS are provided sub-optimally due to a lack of resources. This study sought to examine pharmacy professionals’ perceptions of the future of PMHS.

**Methods:**

Participants comprised pharmacy professionals from NHS Trusts in England that provided a PMHS. Invitations to participate in a qualitative survey and then an interview were sent to pharmacy services at all NHS Trusts that provided a PMHS. This resulted in 100 survey participants and 34 interview participants. Data were analysed using Braun and Clarke’s inductive reflexive thematic analysis.

**Results:**

Two themes were generated: *Enhancing value for service users* and *Improving* e*fficiency*. *Enhancing value for service users* identifies pharmacy professionals’ suggestions for improving the value of PMHS for service users. These include providing access methods extending beyond the telephone, and providing patients/carers with post-discharge follow-up calls from a pharmacist to offer medicines-related support. *Improving* e*fficiency* identifies that, in the future, and in line with NHS plans for efficiency and shared resources, PMHS may become centralised or provided by community pharmacies. Centralised services were considered to likely have more resources available to provide a patient medicines information service compared to hospital pharmacies. However, such a change was perceived to only increase efficiency if patient information can be shared between relevant healthcare settings.

**Conclusions:**

PMHS are perceived by pharmacy professionals as likely to become centralised in the future (i.e., provided regionally/nationally). However, such change is dependent upon the sharing of patients’ information between hospitals and the centralised hub/s or pharmacies. To enhance the value of PMHS for service users, providers should consider establishing other methods of access, such as email and video consultation. Considering the uncertainty around the future of PMHS, research should establish the best way to support all patients and carers regarding medicines following hospital discharge.

## Background

Patient medicines helpline services (PMHS) are available at some National Health Service (NHS) Trusts^1^ in England with the intention of supporting discharged patients whose medicines regimens may have changed during admission. Such services are needed because research suggests that there is a lack of knowledge about medicines among discharged hospital patients in the UK [1], who may not have received important medicines information [2–5]. Additionally, up to 40 % of patients who have been discharged from hospital may subsequently experience medicines-related problems or need support with their medicines [6–9]. For example, a recent systematic review found that more than a third of calls to PMHS involve medicines-related errors [10]. In addition to the impact upon patients themselves, post-discharge medicines-related issues pose a burden for healthcare organisations. Two studies conducted in the UK found the percentage of readmissions that were medicines-related to be 21 % and 38 % [9, 11].

Despite the need for providing patients with medicines-related support post-discharge, a 2017 survey of NHS Trusts found that only 52 % provided a PMHS, and that considerable variation exists in the operation of these services [12]. Additionally, the study showed that the access, availability, and promotion of PMHS were not meeting Royal Pharmaceutical Society-endorsed national standards regarding the provision of PMHS [12, 13]. A recent qualitative study examined pharmacy professionals’ experiences and perceptions of providing PMHS [14]. The study found that, although PMHS are considered by pharmacy professionals to have a number of benefits for service users, services are perceived to be under-resourced (i.e., no specific funding, understaffed), which limits their overall impact [14]. However, a recent systematic review examining the effectiveness of PMHS, and a qualitative study of experiences of contacting a PMHS, found that these services are usually valued by their users [15, 16]. Various positive outcomes after consulting a PMHS are also reported by service users, including improved health, reassurance, and resolution or avoidance of problems [15, 16]. Therefore, PMHS provide an important and valued function, although their provision could be significantly improved.

Recent developments within the NHS, such as the Five Year Forward View [17, 18], the Carter Report [19], and the NHS Long-Term Plan [20], recommend that NHS organisations within regions of England collaborate by sharing services and resources to a greater extent in the future. Sharing services and resources across NHS Trusts within regions may provide a solution to the current suboptimal provision of PMHS. Additionally, the new community pharmacist contract for England has outlined plans for an essential medicines reconciliation service, the NHS Discharge Medicines Service, to come into effect within the next year [21, 22]. This service will make the transfer of patients’ discharge information from NHS Trusts to community pharmacies easier, which could mean that, aside from hospital pharmacists, community pharmacists also have the information required to answer medicines-related questions from patients and carers following their discharge from secondary care. However, to date, no study has sought to elicit the views of pharmacy professionals’ who provide PMHS specifically regarding the future of this service, and the feasibility of sharing services and resources within regions. This novel study therefore aimed to explore pharmacy professionals’ views regarding the future of PMHS using qualitative methods, and to make recommendations to improve the provision of medicines information (MI). The following research question was addressed: *What are pharmacy professionals’ views regarding the future of NHS patient medicines helpline services?*

## Method

### Study design

This study implemented an online qualitative survey design, with subsequent follow-up semi-structured telephone interviews. The authors adopted the epistemological position of pragmatism [23].

For transparency, the study authors comprise three University academics with PhDs (one male, two female) and one PhD student with an MSc (male). All study authors have an interest in pharmacy practice, health services research, and/or health psychology. One author (MJ) has prior experience of managing a PMHS for an NHS Trust in England.

### Participants and recruitment

For both the qualitative survey and the interview, eligible participants were pharmacy professionals (registered with the General Pharmaceutical Council) at an English NHS Trust that provided a PMHS (i.e., Chief Pharmacists, pharmacists, and pharmacy technicians were all eligible to participate).

A relationship was not established with potential participants prior to study commencement, and contact was first made with the invitation to participate. The invitation to participate informed participants of the researchers’ aims of the research (i.e. to make recommendations to improve PMHS by exploring the views and experiences of pharmacy professionals). For the survey and interviews, invitations were sent by email to all NHS Trusts that provided a PMHS in England (*n* = 117 Trusts, [12]), with both a pharmacy professional who provides the Trust’s PMHS and the Trust’s Chief Pharmacist eligible to participate. For the survey, this resulted in 100 participants from 86 Trusts; for the interviews, this resulted in 34 participants from 34 Trusts (none dropped out). This number of interviews is congruent with recommendations in the literature for similar studies [24].

The concept of data saturation was not used to determine the sample size, as this approach has been challenged [25]. Concerns include the suggestion that, because there might always be potential codes and themes to identify, true saturation may not exist [26].

### Data collection

Survey data were collected from February to May 2017, and interview data were collected from May to October 2018 by MW, who had been trained to conduct research interviews. The data collection procedures have previously been published elsewhere [12, 14]. A qualitative survey was chosen so that a large number of pharmacy professionals could participate, in order to collect a diversity of in-depth views pertaining to the research question [27]. One-to-one semi-structured interviews were subsequently selected in order to collect a deeper level of data to that of the qualitative survey, by enabling greater flexibility through the use of probes and unplanned questions. For the survey, an online platform was chosen (SurveyMonkey; [28]) for ease of data collection and to encourage good response rates from a group of participants who frequently communicate electronically. To enable the inclusion of participants from a wide geographical area, interviews were carried out by telephone [29]. Participants were interviewed once. During data collection, all participants were allocated a unique identification number (e.g., Survey P1, Survey P2, etc; Interview P1, Interview P2, etc.).

The survey and interview schedule were developed for the purpose of surveying and interviewing pharmacy professionals, and have previously been published elsewhere [12, 14]. Contained within the survey and the interview schedule was the question “How do you see patient medicines helplines at NHS Trusts developing in the future?”. The study authors developed the survey and the interview schedule following established conventions for these research techniques [30–33]. All interviews were audio-recorded and subsequently transcribed verbatim. Transcripts were not returned to participants for comment and/or correction.

### Data analysis

Data from the survey and interviews were analysed using Braun and Clarke’s inductive reflexive thematic analysis (TA) [34]. This transparent technique has been previously used for similar studies because it is a systematic and rigorous approach to organising, describing, and interpreting data [35–38]. Braun & Clarke’s approach to TA was selected because it is theoretically flexible but still gives a step-by-step process for conducting a thorough and transparent analysis.

Following Braun and Clarke, the analysis used the following stages: familiarisation with the data, generating initial codes, developing themes, reviewing themes, defining and naming themes, and writing the analysis [24]. Initial codes were handwritten onto individual survey responses and interview transcripts (by MW), with both data sources coded simultaneously. The development and reviewing of themes were conducted in Microsoft Excel. This was an inductive and iterative process, with repeated reference to the initial codes and the raw data. The findings were not returned to participants for comment.

### Enhancing the quality of qualitative research

This study meets the criteria for demonstrating the quality of qualitative research outlined by Yardley [39]. For *sensitivity to context*, previous literature was reviewed, and we invited participants from all PMHS-providing NHS Trusts. For *commitment and rigour*, TA stages were followed in accordance with Braun & Clarke’s recommendations [24], and there was agreement of analyses among the study team. The consolidated criteria for reporting qualitative research (COREQ) and the standards for reporting qualitative research (SRQR) were also followed [40, 41]. For *coherence and transparency*, the study results are grounded in example quotations from the raw data, and a ‘paper trail’ approach was used. This included the recording of thoughts about each interview in a reflective diary, including its contextual features, and how subsequent interviews might be improved. Use of the study findings to develop recommendations for improving PMHS demonstrates *impact and importance.*

## Results

Table 1 provides an overview of participant characteristics, showing that most were employed within acute NHS Trusts.

**Table 1:** Participant characteristics

Characteristic		Survey participants (*n* = 100)	Interview participants (*n* = 34)
		n (%)	n (%)
Pharmacy professional type	Chief Pharmacist	48 (48 %)	4 (12 %)
	Pharmacist	47 (47 %)	29 (85 %)
	Pharmacy technician	1 (1 %)	1 (3 %)
	Not stated	4 (4 %)	0 (0 %)
NHS Trust type	Acute	82 (82 %)	26 (76 %)
	Mental health	12 (12 %)	3 (9 %)
	Community	3 (3 %)	1 (3 %)
	Specialist	2 (2 %)	2 (6 %)
	Integrated (two or more types)	1 (1 %)	2 (6 %)

*Note*. Abbreviations: *NHS* National Health Service.

Two themes were generated from the analysis: *Enhancing value for service users* and *Improving efficiency*. *Enhancing value for service users* identifies pharmacy professionals’ suggestions for improving the value of PMHS for people who use the service. These include providing additional access methods other than the telephone, and providing patients with post-discharge follow-up calls from a pharmacist to offer support with their medicines. *Improving* e*fficiency* identifies that, in the future, PMHS may become centralised or provided by community pharmacies, with centralised services considered to likely have more resources available to provide this service compared to hospital pharmacies (i.e., funding and staffing). However, such a change may only lead to an efficient system if patient information can be shared between the hospital and whoever becomes the patients’ point of contact.

### Enhancing value for service users

In this theme, participants described how PMHS provide value to service users, and that such services will be increasingly needed in the future as medicines regimens increase in complexity due to an ageing population. Some suggested that this service should be available to *all* individuals, not just discharged patients and their carers.

Solutions were offered for developing PMHS further, in order to enhance the value for service users. Ideas included expanding the hours of the service (e.g., offering the service during evenings and/or weekends, or putting the phone through to the dispensary out-of-hours), improving the advertising of the service (e.g., free advertising, such as in patient discharge summaries, on hospital waiting room monitors, and having healthcare professionals tell patients about it), and using satisfaction surveys in order to establish how their patients would like the PMHS to be improved.

*“I wouldn’t know where we can go next with it [PMHS], without knowing what they [patients] need.”* (Interview, P29).

Several participants spoke of the future need to provide an active service, so that patients who are considered at-risk (e.g., those on complex medicines regimens following an admission) will receive a follow-up phone call from a hospital pharmacy professional within a few days of discharge to check if they require any support with their medicines. There was an acknowledgement among many participants that PMHS do not reach all patients who require support with their medicines, and that many are likely being missed.

*“It’s only patients with a really burning question that would contact us… Or they might not have thought about it… It might be better if we’re a bit more active to ask patients if they’re ok once they’ve gone home."* (Interview, P31).

Another widely considered option for improving PMHS in the future concerned offering alternative contact methods to the telephone (e.g., email, text, face-to-face, online chat, video consultations, apps, and social media such as Twitter and Facebook). Participants described the importance of ensuring that PMHS embrace new technologies, and that they are easily accessible in order to meet differing needs. Reasons why alternatives to the telephone are needed include having options for people who find using the telephone difficult (e.g., communication difficulties; inability to call during the day) and to widen the appeal of the service (e.g., “*Extension towards social media and/or Skype etc. to widen access to younger people*.”; Survey, P80). Importantly, alternatives to telephone provision for PMHS where a visual is also provided may facilitate improved communication (e.g., the use of non-verbal communication, and the ability to see a patient’s medicines). This is exemplified in the following quotation.

“*I quite like the idea of having Skype, because that allows you to actually communicate better with a patient, if you’re able to assess their physical communication. You lose a significant proportion of communication just by doing it over the phone or over email*.” (Interview, P34).

Participants also spoke of making improvements to existing access options, such as ensuring that their email address is added to promotional material, and making their webform easier to find via the Trust website. However, some participants were concerned about confidentiality issues surrounding some of these access methods, which may impede their implementation.

### Improving efficiency

Participants described how, in the future, PMHS are likely to become centralised (e.g., provided regionally or nationally) rather than remaining local to hospitals, since commissioners may see this as a more cost-efficient model. Some participants perceived this to be an improvement to the current localised system due to improved cost-effectiveness.

*“We would like to see a national generic medicines helpline with local queries referred to the trust concerned, as this would be more cost effective.*” (Survey, P54).

However, most participants believed that PMHS should remain local to hospitals, since hospital resources are needed to answer questions pertaining to hospital treatment (e.g., drug charts, inpatient notes, blood results, access to specific consultants or other HCPs, errors specific to that hospital, Trusts’ policies/procedures). Participants suggested that the centralisation of PMHS could be a viable option if the sharing of patient information was greatly improved across the relevant organisations within the NHS.

*“If we all become electronic, if we all share the same system, and if we all read the same thing when we’re looking at the screen, then it might work.“* (Interview, P5).

Without such improvement, enquiries needing local knowledge would require a call to the relevant Trust to request the information, and such a delay was perceived to be an inefficient system. There was also concern that centralisation would be an impersonal service, which could decrease service user satisfaction. Linking to the first theme, this is an example of how striving for efficiency may result in a decrease in value.

*“You wouldn’t get that sort of personal touch of being able to speak to someone who actually may have been on the ward that they were discharged from… So I certainly think from a patient point of view, it might lose some of the quality.”* (Interview, P32).

Additionally, participants from mental health and specialist services were concerned that their patients would need to speak to a pharmacy professional with such specialist knowledge, which may not be possible at a centralised hub.

Perceived positive outcomes of centralisation were the coordination and standardisation of the service within the region to reduce unnecessary duplication, reducing the workload of hospital pharmacists so that more time could be spent on discharge counselling, and having a service that may be better resourced. For example, if specific funding was allocated for the service, this would improve its promotion and hours of availability, which could mean that more service users are able to obtain support.

*“[With a centralised service], they [enquirers] would get a bit more reception... We run the MI service [at the hospital] but we’ve also got ward commitments as well. So, a central centre I suppose wouldn’t have those commitments.”* (Interview, P31).

Participants suggested some examples of how a centralised model could work, if patient information could be shared. For example, NHS 111, the NHS telephone and online advice service [42], was suggested as an option for the centralised service, provided that enquiries were answered by pharmacy professionals with expertise in MI. Another option was for community pharmacies to be the initial contact point for MI enquiries, with support from local hospital pharmacy services when needed.

*“What I’d really like to see is a networking, or triaging sort of situation, where community pharmacies are the first port of call, and then we back them up if they can’t deal with it.”* (Interview, P18).

Another option for a more efficient system was to ensure better join-up of primary and secondary care services regarding the patient information shared between them. It was suggested that primary care and community services should be informed of enquiries to the hospital PMHS, should the patients subsequently seek their services. This will help to ensure that healthcare professionals involved in patients’ care can access relevant information pertaining to patients’ prior contacts with healthcare services.

*“We might’ve answered a question from a patient; maybe then we need to be putting that on to their GP record or something. Or on to somewhere. I mean, we document it, but no-one else sees it.”* (Interview, P11).

## Discussion

This multi-method study explored the views of pharmacy professionals regarding the future of PMHS. Using qualitative survey and interview data, two themes were generated: *Enhancing value for service users*, and *Improving efficiency*. The findings illustrate pharmacy professionals’ views as to how the value of PMHS for patients and carers might be enhanced in the future. These include providing additional access methods other than the telephone, and providing an active service where patients receive a post-discharge call from a pharmacy professional from their hospital to establish if they require medicines-related support. Pharmacy professionals also believed that, in line with NHS plans for efficiency and shared resources, PMHS may become centralised in the future. However, many participants expressed concerns that such a change will be an inefficient system if patient information cannot be shared between hospitals and the centralised hub/s, and that this may result in a service that has less benefit and value for patients and carers compared with the current provision of hospital-based PMHS. Another suggestion was for community pharmacies to become the initial point of contact for MI queries after hospital discharge, with support from hospitals when needed.

Our findings suggest that one potential way to improve PMHS is to provide additional access methods other than the telephone. This accords with a qualitative study of service users’ experiences of PMHS, which suggested that enquirers would prefer to access the service via telephone, but that other options (e.g., email, apps, and video consultations) might be more appealing to younger individuals [15]. However, the preference for telephone access may be unsurprising in that study’s sample, given that the average age of participants was 68 years, and that all participants were chosen because they opted to contact a PMHS (i.e., a telephone helpline).

The 2019 NHS long-term plan mandates that patients and carers will have access to healthcare services online via video consultations within five years [20]. Additionally, the Covid-19 pandemic may accelerate the use of video consultations for healthcare services that do not require attendance at a healthcare setting [43]. Research suggests that there is a demand for video consultations from patients [44, 45]. For example, a recent qualitative interview study by Donaghy et al. explored the acceptability, benefits, and challenges of video consultations in primary care, between patients and clinicians [45]. They found that patients and clinicians considered video consultations as superior to telephone consultations regarding reassurance, building rapport, and communication, as a result of having visual cues. However, technical issues were common, and the technical infrastructure required to allow video consultations to become routine was considered a significant barrier. Relatedly, Hammersley et al. conducted a non-randomised, quasi-experimental study to compare the content, quality, and experience of video consultations, telephone, face-to-face consultations with GPs [46]. The study found that both video consultations and telephone consultations were comparable in terms of content and quality. Participants chose the access method they preferred, with video consultations being chosen by younger patients. Providing MI via video consultation may therefore be a method for attracting younger patients to seek support, since studies show that most callers to PMHS are aged 65 and over, with an average age of 69 years [10, 47–49].

Our findings highlight that, although providing a PMHS is considered to be a useful tool for helping those patients and carers who choose to seek support with their medications, a gap exists for supporting those patients and carers who, for whatever reason, do not seek support (e.g., they may not realise that an issue exists). Several participants in this study proposed that a future initiative could be for NHS Trusts to also provide a pro-active service, whereby patients receive a post-discharge follow-up call from a pharmacy professional of the hospital shortly after the patient’s discharge. To date, little research has been conducted to examine the effectiveness of such a service. A prospective cohort study conducted in 2013 at two hospitals in the UK found that telephone follow-up calls to patients within 14 days of discharge were effective at solving or avoiding medication problems [50]. Additionally, out of 62 participants in the study, 75 incidents were identified that could have caused harm to patients (49.3 % rated as moderate risk, 29.3 % as high risk, and 9.3 % as extreme risk). However, compared to a control group, the intervention did not show a reduction in 30-day hospital readmissions. Although a larger-scale, randomised trial is needed to establish its effectiveness and cost-effectiveness in terms of readmissions, these findings highlight that patients do benefit from a telephone follow-up call intervention post-discharge from hospital. A study examining pharmacy professionals’ experiences of providing a PMHS found that helplines are often provided with limited resources [14]. Therefore, although providing a proactive post-discharge MI service is likely to be of value to patients, NHS trust will need to allocate additional resources in order to support such a service.

Our findings also show that pharmacy professionals believe that PMHS are likely to become centralised in the future (e.g., provided regionally or nationally). This reflects recent developments within the NHS, such as the Five-Year Forward View [17, 18] and the NHS Long-Term plan [20], which aim to develop Integrated Care Systems across regions of England by April 2021 for the efficient use of resources and the design of services. However, participants also believed that the quality of centralised PMHS is likely to be compromised if the centralised hub/s do not have access to all of the required patient information. This was the main reason for participants believing that PMHS should remain provided by hospitals for their own patients. This accords with a recent study by Badiani et al. who found that 75 % of 200 enquiries to a PMHS could only be addressed with access to local knowledge such as medical records and healthcare professionals involved in the patient’s care [51].

In the current study, pharmacy professionals suggested that, in the future, there could be greater collaboration with NHS 111 and primary care. An example was having community pharmacies as the initial point of contact for post-discharge MI queries, with backup from local hospitals where necessary. The new community pharmacy contract for England has outlined plans for a medicines reconciliation service, the NHS Discharge Medicines Service, to come into effect within the next year [21, 22]. This builds upon the Transfers of Care around Medicines (TCAM) services that some NHS Trusts have been providing since 2014 [52]. TCAM enables NHS Trusts to send referrals and patient discharge documentation to nominated community pharmacies regarding patients that need additional support with their medicines after leaving hospital. The community pharmacy is then able to accept or reject the referral, and if accepted, will contact the patient for a post-discharge support consultation. Thus, such a service would function as providing post-discharge follow-up calls to at-risk patients, as proposed by study participants, albeit by community pharmacists rather than hospital pharmacists. An evaluation of one TCAM, published in 2020, found that patients followed up by a community pharmacist after being discharged from hospital were significantly less likely to be readmitted to hospital within thirty days [53]. However, this was not a randomised controlled trial; it was an observational study comparing referred patients who were accepted for intervention compared to referred patients who were not accepted for intervention. Therefore, the groups were not even matched. This study design is typical of the available evidence pertaining to the TCAM scheme [54–56], and so, higher quality research is needed to establish its effectiveness for reducing readmissions (i.e., a randomised controlled trial). Based upon the limited available evidence to date, it has been estimated that TCAM saved the health economy more than 50 million pounds in 2018–2019, by reducing the number of hospital readmissions [52].

Since NHS England is aiming to roll out the TCAM scheme to all acute Trusts [52], the transfer of patients’ admission and discharge information to community pharmacies could mean that community pharmacists may be in a position to access the information required to answer medicines-related questions from patients and carers following their discharge from secondary care. However, this would require *all* hospital patients’ admission and discharge information being available to a nominated community pharmacy, and not just those patients deemed to be high-risk, as is the current remit of the TCAM service. Therefore, the TCAM scheme would need to be extended to be applicable to *all* patients. Additionally, since TCAM currently only applies to acute Trusts, it seems likely that non-acute Trusts (i.e., mental health, specialist, and community Trusts) would need to provide their own MI services to their patients. However, findings from a survey conducted in 2017 revealed inequity in the provision of PMHS, as the proportion of mental health and community Trusts able to provide such services (29% and 18% respectively) was less than half that seen for acute Trusts (67%) [12].

Although evidence suggests that many PMHS are provided with limited resources and that they do not meet national standards for providing a PMHS [12, 14], as described above, the provision of MI to patients and carers following patients’ hospital discharge by community pharmacists is also likely to be limited. Currently, the pharmacy systems pertaining to secondary care and primary/community care exist largely independently and without reference to one another, and all provide services within resource limited contexts. For example, studies pertaining to other enhanced community pharmacy initiatives, such as Medicines Use Reviews and New Medicines Service, suggest that community pharmacy staff view providing such additional services as increasing their workload in an already pressured, time-limited environment [57–64]. Thus, rather than having a system of multiple inadequately resourced ways for patients and carers to obtain medicines-related support following hospital discharge (e.g. PMHS and TCAM), co-ordinated thinking is needed to explore how best to provide such support to all patients.

### Recommendations for practice

A working group of relevant stakeholders pertaining to PMHS and the TCAM service (e.g., hospital pharmacists, community pharmacists, patients and carers) should be formed to discuss and establish how PMHS and TCAM fit together, so that duplication of effort is avoided whilst at the same time ensuring that all patients and their carers are supported regarding medicines.

Providers of PMHS should consider additional methods of accessing their service besides the telephone, and to ensure that all methods of access are stated on the service’s promotional materials.

### Recommendations for future research

Currently, approximately half of NHS Trusts (mostly acute) provide a PMHS, and considerable variation exists regarding the services’ hours of availability across Trusts [12]. The present study also highlights that PMHS cater only to individuals seeking medicines-related support, and does not reach those patients and carers who, for whatever reason, do not seek such support. A useful research avenue would therefore be to explore the views of relevant stakeholders (e.g., service users, pharmacy professionals, service commissioners) regarding how best to provide medicines-related support to *all* patients and carers following hospital discharge.

Another research endeavour could be to conduct a cluster randomised controlled trial to establish the effectiveness and cost-effectiveness of providing post-discharge follow-up calls to patients from a pharmacy professional within two weeks of discharge from hospital. The intervention would offer support with medicines, to establish whether this results in reduced hospital readmissions and visits to other services (e.g., primary care). Patients considered most in need of support could be targeted (e.g., those discharged on complex medicines regimens).

### Strengths and limitations

This is the first study to explore pharmacy professionals’ views of the future of PMHS. A multi-method approach was used, thereby providing diverse and rich data that can contribute to recommendations for future research and service improvements.

A limitation of this study is that the perspectives of pharmacy professionals who chose not to participate are not represented. In addition, the sample would have been improved with more participants from mental health, specialist, and community Trusts. Relatedly, the study could have been improved by the addition of perspectives of pharmacy professionals who do not provide a PMHS, from other healthcare professionals (e.g., GPs, nurses, physicians), and also patients and carers.

## Conclusions

This multi-method qualitative study highlights pharmacy professionals’ views as to how to enhance the value of PMHS, namely providing additional access methods other than the telephone, and providing post-discharge follow-up calls to patients. Additionally, pharmacy professionals also believed that, in order to aim for efficiency, PMHS are likely to become centralised in the future (i.e., provided regionally or nationally). However, such a change was perceived to be largely inefficient if patients’ information could not be shared with the centralised hub. We recommend that helpline providers consider providing other methods of access, such as email and video consultation, and that all access methods are stated on promotional materials. Since there is uncertainty as to the future of PMHS, further work is required to establish the best way to support all discharged patients with their medicines.

## Data Availability

Survey data are not available, since a data access statement was not included in the participant consent form. Anonymised interview transcripts and datasets are available from the University of Bath Research Data Archive to bona fide researchers [65].

## Abbreviations

GP: general practitioner
MI: medicines information
NHS: National Health Service
PMHS: patient medicines helpline service
TCAM: Transfer of Care Around Medicines
UK: United Kingdom

## Footnotes

1 The English National Health Service (NHS) is organised in to NHS Trusts, which are organisations that provide goods and services for the purposes of health care (e.g., hospital and community services), and each Trust primarily serves a geographical area within England.
